# Postinfectious Syndromes and Long-Term Sequelae after *Giardia* Infections

**DOI:** 10.3201/eid3114.241793

**Published:** 2025-12

**Authors:** Shanna Miko, Pallavi A. Kache, Erin Imada, Amy L. Freeland, Julia C. Haston

**Affiliations:** Centers for Disease Control and Prevention, Atlanta, Georgia, USA

**Keywords:** Giardia, parasites, syndromes, sequelae, postinfectious irritable bowel syndrome, infection-associated chronic illness, chronic fatigue, fibromyalgia

## Abstract

Giardiasis, caused by the parasite *Giardia duodenalis*, is a common infection throughout the world. Acute infections can be asymptomatic, cause mild gastrointestinal symptoms, or be associated with severe, prolonged diarrhea. Most *Giardia* infections are self-limiting; however, a subset of symptomatic and asymptomatic persons experience infection-associated chronic conditions that can affect multiple body systems. Those conditions include stunting and impaired cognitive function in children, irritable bowel syndrome, chronic fatigue, arthritis, and fibromyalgia, all of which can persist for months or years. Such conditions can impair daily functioning and quality of life; however, research has yet to fully elucidate underlying mechanisms, describe the prevalence, identify persons at increased risk, and develop effective treatment strategies. We synthesized what is known about giardiasis-associated chronic conditions and illnesses to improve recognition of those complications and ensure appropriate management that can improve the well-being of persons affected.

Infection with the parasite *Giardia duodenalis*, known as giardiasis, is reported throughout the world, including in the United States (https://www.cdc.gov/giardia). *G. duodenalis* infection is endemic in many regions, and prevalence estimates range from 3%–10% in high-income countries to 20%–35% in low-income countries (*1*,*2*). In the United States, *G. duodenalis* is the most common intestinal parasitic infection among humans (*3*). The pathogen is transmissible through multiple pathways, including contaminated water or through the fecal–oral route. Infections are associated with poor sanitation, human-to-human or animal-to-human spread, and exposure to untreated or inadequately treated fresh water by drinking or swimming (*2*). Acute giardiasis symptoms vary across world regions and include severe, prolonged diarrhea, mild or intermittent gastrointestinal illness, and extraintestinal manifestations, whereas some infections can be asymptomatic (*2*). Asymptomatic infections in children of endemic areas can be a major contributor to complications later in life (*4*,*5*). Most *G. duodenalis* infections are self-limiting, but some symptomatic and asymptomatic persons experience infection-associated chronic conditions and illnesses (IACCIs). IACCIs can affect multiple body systems and persist for months or years, resulting in a disease burden that greatly reduces daily functioning and quality of life (*2*,*6*,*7*).

Some IACCIs, such as functional gastrointestinal disorders (FGIDs) and myalgic encephalomyelitis or chronic fatigue syndrome, may be reported after infections including, but not limited to, COVID-19, Lyme disease, campylobacteriosis, and giardiasis (*7*) (https://www.cdc.gov/chronic-symptoms-following-infections). Data collection for IACCIs remains challenging, given the multiple pathophysiologic mechanisms, varied symptomatology, and wide-ranging period of syndrome onset and duration. Therefore, prevalence and risk factors for the conditions are not fully understood (*7*). Giardiasis increases intestinal epithelial permeability, resulting in malabsorption and mucus depletion, and may induce intestinal dysbiosis. This intestinal barrier disruption is believed to be associated with some IACCIs (*2*,*8*). Early diagnosis could mitigate the most severe disability symptoms and optimize outcomes among patients (*7*,*9*).

We have synthesized what is known about the clinical manifestations of IACCIs after giardiasis. We focused on IACCIs most often reported in the literature, including FGIDs, musculoskeletal and neuromuscular syndromes, and pediatric implications such as stunting and cognitive impairment (Figure). We then identified knowledge gaps in the epidemiology of IACCIs after giardiasis in the United States, including the defining of high-risk populations. We explored potential approaches to expand our understanding of these IACCIs through routine analyses of national hospitalization databases, insurance claims databases, and active surveillance programs. Our discussion aims to support public health efforts around IACCIs after giardiasis and contribute to an increased understanding of these conditions more broadly. Increasing healthcare provider (HCP) awareness around IACCIs is essential in providing timely and targeted treatment and improving patient outcomes.

**Figure:**
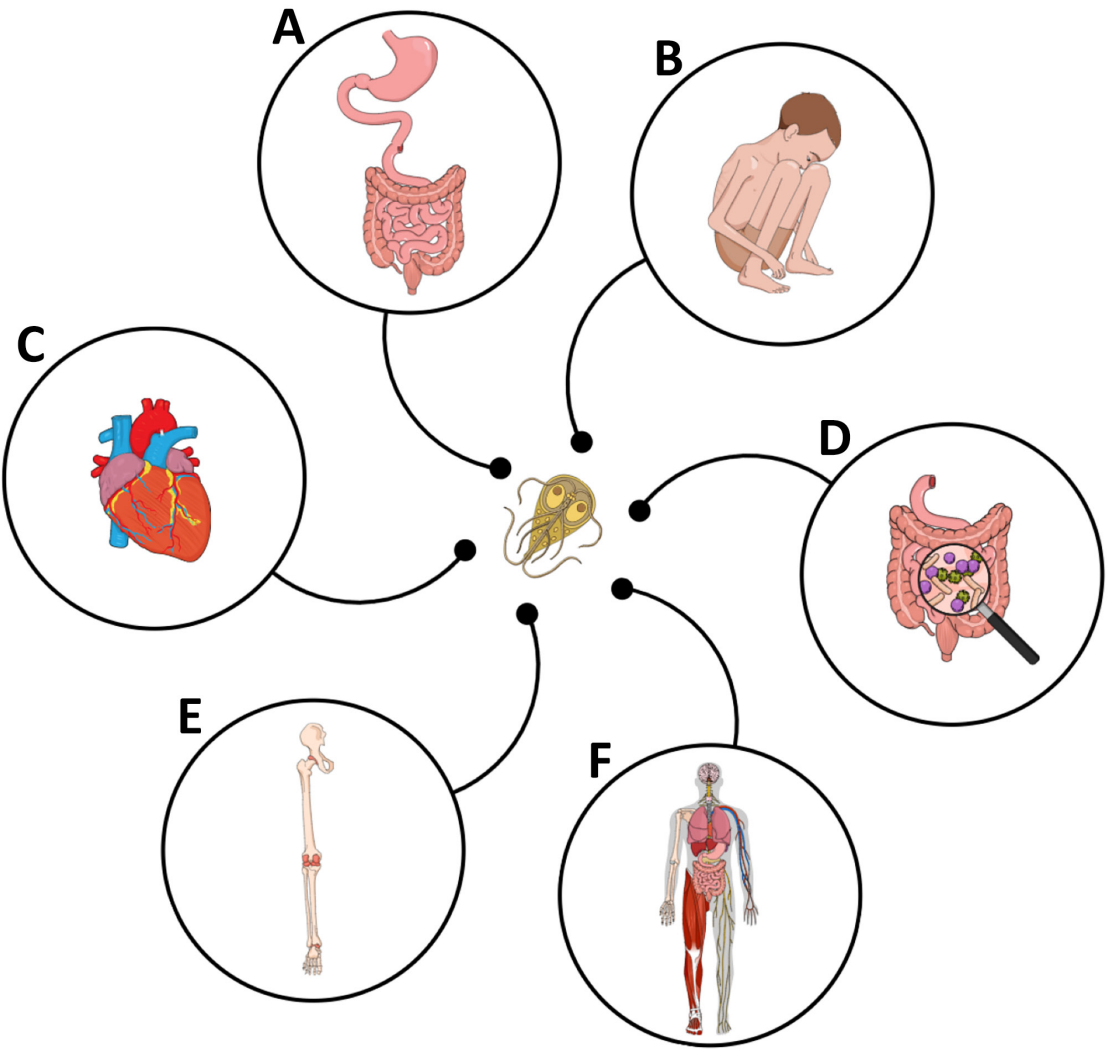
Clinical manifestations of infection-associated chronic conditions and illnesses most often reported after giardiasis. A) Postinfectious irritable bowel syndrome, postinfectious functional dyspepsia, gastresophageal reflux, bloating, and nausea. B) Cognitive impairment, failure to thrive, and stunting. C) Myocarditis. D) Treatment-refractory giardiasis. E) Arthridites. F) Fibromyalgia and myalgic encephalomyelitits/chronic fatigue syndrome.

## Approach to Summarizing the Literature

We reviewed Ovid and Scopus databases in May 2024 to identify studies in English without date restrictions. We used Medical Subject Headings terms alone or in combination to capture complications of giardiasis infections in humans, including persistent and chronic infections, chronic symptoms, postinfectious syndromes, and long-term sequelae. We included articles if the research or report involved human subjects, were published in peer-reviewed journals, had a full text or abstract in English, and included evidence of postgiardiasis chronic sequelae. We excluded articles if they were written without original peer-reviewed data, written in languages other than English, or involved animal models. We identified duplicates by using the Endnote (https://web.endnote.com) automated “find duplicates” function, set preferences to match on title, author, and year, and removed duplicates from the Endnote library. Of the 3,135 initial records, we removed 1,542 (49%) duplicate results, 842 (27%) publications that excluded postgiardiasis chronic sequelae, 375 (12%) studies not written in English, and 194 (6%) animal model studies, leaving 182 publications. Although the quality of data among the articles varied, we attempted to include publications about all proposed IACCIs to describe the breadth of IACCIs that may be associated with giardiasis. We also summarized studies and reviews identified in the search but not included in this article (Appendix).

## Postinfectious FGIDs

Chronic gastrointestinal symptoms associated with giardiasis are most often described in the literature. Postinfectious FGIDs (PI-FGIDs) involve recurring or chronic gastrointestinal symptoms without other known disease processes. They are frequently classified by using Rome criteria for FGIDs, which do not necessarily occur after infections (*10*,*11*). The Rome IV criteria consists of 33 adult and 20 pediatric FGIDs, defined on the basis of the type, duration, and frequency of gastrointestinal symptoms (https://theromefoundation.org/rome-iv/rome-iv-criteria). Two frequently diagnosed giardiasis-associated PI-FGIDs are postinfectious irritable bowel syndrome, often clinically indistinguishable from irritable bowel syndrome (IBS) not associated with infection, and postinfectious functional dyspepsia (*10*).

## Postinfectious IBS

Postinfectious IBS (PI-IBS) symptoms commonly include diarrhea or diarrhea alternating with constipation, bloating, and recurring abdominal discomfort for >6 months (*8*,*11*,*12*). Reports highlight bloating, diarrhea, nausea, and abdominal pain as the most severe symptoms (*11*,*13*). In a study of 82 persons with giardiasis-associated PI-FGIDs (81% of whom had PI-IBS), symptom exacerbation was related to specific foods (58% of cases) and physical or mental stress (45% of cases) (*11*). The foods associated with the most complications included dairy products, vegetables, fruit, alcohol, and foods from fermentable oligosaccharides, disaccharides, and monosaccharides and polyols subgroup polyols (i.e., sugar alcohols) (*14*). Other PI-IBS symptoms included gastric hypersensitivity, decreased drinking capacity, and reduced gastric emptying (*15*). Regardless of symptoms, many persons with giardiasis-associated PI-IBS reported a negative effect on their quality of life associated with the IBS and fatigue symptoms (*6*).

Robust evidence supporting the relationship between giardiasis and PI-IBS comes from studies regarding 2 separate drinking water contamination events in northern Europe. The research demonstrated an increased risk for IBS symptoms after giardiasis or cryptosporidiosis, on the basis of Rome criteria, at 3 (adjusted risk ratio [aRR] 3.4; 95% CI 2.9–3.8) (*16*), 6 (aRR 3.4; 95% C: 2.9–3.9) (*17*), and 10-year intervals postinfection (adjusted odds ratio 4.7; 95% CI 3.6–6.2) (*18*). Follow-up studies noted a small but significant decrease in the prevalence of PI-IBS from 3 to 6 years but no change from 6 to 10 years, indicating that symptoms may improve for some patients but can have long-lasting effects for others (*17*,*18*). Another drinking water contamination event further demonstrated this association; persons who were affected by giardiasis, campylobacteriosis, or norovirus had a nearly 3-fold (odds ratio [OR] 2.6; 95% CI 1.4–1.7) increase in the odds of IBS onset 1 year later compared with persons who were not infected (*19*).

PI-IBS is the most common PI-FGID, occurring in an estimated 10%–20% of persons after gastrointestinal infection (*8*,*10*–*12*,*20*–*23*). A previously healthy person recovering from an acute gastrointestinal infection, regardless of pathogen type (e.g., viral, bacterial, or parasitic infection), has a 3- to 4-fold increase in risk for IBS symptom onset compared with unexposed persons (*8*,*24*). One study analyzed data from a commercial insurance database and reported that the 1-year incidence of IBS was higher in persons with previous giardiasis (37.7/1,000 person-years) compared with persons without a documented *G. duodenalis* infection diagnosis (4.4/1,000 person-years) (*23*). After adjusting for anxiety, depression, and healthcare use, the adjusted hazard ratio was 3.9 (95% CI 2.9–5.4) (*23*). Similar outcomes were observed in a pooled analysis of several cohort studies, where documented giardiasis infection increased the odds for developing IBS >5-fold (OR 5.5; 95% CI 4.2–7.1) compared with uninfected persons (*25*).

Factors associated with a higher risk for PI-IBS onset include younger age (*8*), female sex (*8*,*12*,*25*,*26*), antibiotic exposure (*25*), severity of initial infection (*8*), prior mental health diagnosis (*8*,*12*,*18*,*19*,*25*), somatization (*19*), and giardiasis infections that require >1 treatment course (i.e., treatment-refractory giardiasis) (*26*). Moreover, data suggest protozoal infections such as giardiasis and blastocystosis carry a higher risk for PI-IBS onset (*16*,*27*) compared with bacterial and viral gastrointestinal infections (*8*,*21*,*25*).

## Postinfectious Functional Dyspepsia

Postinfectious functional dyspepsia (PI-FD) is characterized as an onset of new dyspeptic symptoms after an acute gastrointestinal infection (*10*). Symptoms can include constipation, epigastric pain, severe bloating, gastric hypersensitivity, decreased drinking capacity, and reduced gastric emptying (*11*,*15*). Independent factors associated with a higher risk for PI-FD include younger age, somatization (*19*), and prior mental health diagnosis (*27*). Considerable overlap seems to exist between PI-IBS and PI-FD. Some studies note that, among participants meeting the Rome criteria for PI-IBS, a subset (15%–44%) also met criteria for PI-FD (*11*,*13*). Likewise, ≈85% of participants with PI-FD also met Rome criteria for PI-IBS in another study (*13*).

An estimated 10% of persons with gastrointestinal infections might have onset of PI-FD (*23*). A systematic review described increased odds of PI-FD onset 6 months after a gastrointestinal infection (OR 2.5; 95% CI 1.8–3.7) compared with a control population (*23*). Further, some large cohort studies indicated that ≈25% of persons with giardiasis had onset of PI-FD (*11*,*13*). Another cohort study examining active-duty military personnel with prior giardiasis documented an increased risk for PI-FD (risk ratio 3.2; 95% CI 1.2–8.9) compared with controls (*27*).

## Other Gastrointestinal Symptoms

Giardiasis is also a risk factor for developing chronic gastrointestinal symptoms that do not meet PI-IBS or PI-FD criteria. One cohort study described prior giardiasis associated with an increased risk (risk ratio 4.0; 95% CI 2.9–5.6) of gastroesophageal reflux (*27*). Another study reported an increase in the prevalence of bloating (aRR 1.8) and nausea (aRR 3.0) compared with persons without prior giardiasis (*13*).

## Postinfectious Musculoskeletal and Neuromuscular Syndromes

An estimated 30% of persons with prior giardiasis experience long-term extraintestinal symptoms (*28*). Many of those symptoms are expressed as joint or fatigue syndromes, but some persons report a wide variety of symptoms that often overlap with diagnostic criteria for other extraintestinal syndromes. Frequent manifestations include a decrease in daily functional status, exertion intolerance, unrelieved fatigue despite rest or sleep, neurocognitive and sensory impairments, and musculoskeletal complaints. Although some of these symptoms occur in the absence of gastrointestinal symptoms, many co-occur with PI-IBS (*7*,*29*).

## Postinfectious Arthritis

Case reports of postinfectious joint pain and arthritis were once rare; however, since the 2010s, studies increasingly report arthritides after giardiasis (*30*–*34*). Still, the pathogenesis remains unclear, and no standardized timeframe is used to diagnose the condition (*30*,*33*). Studies use wide-ranging definitions for symptom onset, ranging from >4 days to <3 months after giardiasis and an average duration in joint symptoms of 3 months; however, symptoms can last years (*33*). Postgiardiasis arthritis can manifest in >1 joint, often in the knee, ankle, hip, and wrist (*34*). Research is sparse on identifying risk factors, although a higher risk among children has been reported. One study demonstrated an association between arthritis and previous *G. duodenalis *infection among children 0–19 years of age and adults 20–64 years of age (*33*), and a systematic review reported that the prevalence of postgiardiasis arthritis was higher among children <18 years of age compared with adults (*34*).

An analysis of healthcare encounters and insurance claims in the United States described the odds of arthritis or joint pain within 6 months of giardiasis as 51% higher compared with controls (adjusted odds ratio 1.5; 95% CI 1.3–1.8) (*33*). Despite the high prevalence of giardiasis globally, postgiardiasis arthritis is not diagnosed as frequently as arthritis after other gastrointestinal infections such as *Campylobacter jejuni* (*30*).

## Myalgic Encephalomyelitis or Chronic Fatigue Syndrome

Myalgic encephalomyelitis or chronic fatigue syndrome (ME/CFS) is characterized by waxing and waning physical, cognitive, or emotional exertion intolerance that is not relieved by sleep or rest (*7*). Its pathogenesis remains unclear, and the time to onset varies, but the trajectory is similar. A prodromal phase progresses to a nadir, with some level of improvement (*35*), followed by a chronic phase in which preillness health and abilities are not regained (*9*). Impairments often are associated with extended leave from work and school (*9*,*26*,*36*). Regardless of symptoms, persons with giardiasis-associated ME/CFS report a negative impact on their quality of life associated with disabilities and disruptions of activities of daily living (*6*).

Giardiasis-associated ME/CFS was first reported after a 1986 California water contamination event (*37*). It was again documented after a 2004 water contamination event in Norway (*31*), when ≈5% of persons developed ME/CFS-like symptoms after giardiasis resolution (*36*). Two years after the event in Norway, questionnaire respondents reported more fatigue symptoms than gastrointestinal symptoms (*31*). A follow-up study 3 years postexposure validated the symptoms and identified persons with history of giardiasis as having an increased risk for ME/CFS compared with controls (aRR 4.0; 95% CI 3.5–4.5) (*16*). At 6 years, the fatigue risk decreased slightly but remained higher compared with unexposed persons (aRR 2.9; 95% CI 2.3–3.4) (*17*).

The severity of the giardiasis and having >1 treatment course are associated with ME/CFS (*26*). Chronic fatigue syndrome may also be associated with overactive bladder in both giardiasis-exposed and unexposed persons (OR 2.73, 95% CI 1.85–4.02 in exposed persons; OR 2.79, 95% CI 1.69–4.62 in unexposed persons) (*38*). Throughout follow-up studies, ME/CFS symptoms are associated with IBS symptoms across all age and sex groups (*16*,*17*,*39*).

## Fibromyalgia

Evidence of postinfectious fibromyalgia after giardiasis is emerging. Symptoms of fibromyalgia include chronic generalized pain accompanied with chronic fatigue, disrupted sleep patterns, headache, cognitive dysfunction, and gastrointestinal symptoms (*40*).

A study after a drinking water contamination event in Finland associated with several types of gastrointestinal infections (including giardiasis) described postinfectious complex regional pain syndrome and fibromyalgia in 4% of study participants (*32*). However, the report did not describe associations specifically among persons with giardiasis (*32*). A study from a separate outbreak in Norway detailed a higher prevalence of fibromyalgia (9%) 10 years postgiardiasis compared with persons who had no prior giardiasis (3%) (*29*). The report also described the odds of fibromyalgia were nearly 3-fold among case-patients compared with controls (OR 2.9; 95% CI 1.7–4.9) (*29*).

At the time of this publication, studies have not demonstrated sex or age as risk factors for postinfectious fibromyalgia. However, some analyses reveal that diagnosis often is concomitant with IBS or ME/CFS status after giardiasis (*29*).

## Pediatric Implications

Despite the reduction of intestinal infectious diseases such as giardiasis as leading causes of death in children <5 years of age, they remain a leading cause of disease burden and disability-adjusted life-years globally (*41*). Diarrheal illness in early childhood has been linked not only to impairments in hand–eye coordination, physical fitness, information processing, and cognitive function (*42*,*43*) but also to growth disorders. A study from Ethiopia of 224 children 2–5 years of age demonstrated increased odds of stunting among children infected with >1 intestinal parasites compared with those without (*44*). Furthermore, research shows that giardiasis, even when asymptomatic, is a major contributor to stunting in children (*4*,*5*). Given that a recent study of >11,000 children in low-resource settings identified *G. duodenalis *parasites in stool samples of 35% of asymptomatic children, millions of children worldwide may be at risk for giardiasis-associated stunting (*2*).

Although *G. duodenalis *infections can cause nutrient malabsorption and malnutrition in any human host if not adequately treated, such infections can contribute to IACCIs in children who are still growing and developing (*30*). Persistent giardiasis within the first 6 months of life is associated with deficits in both weight and length at 24 months of age in a large study of children from several countries (*5*). Fecal presence of *G. duodenalis *parasites in 3–6-month-old infants (p = 0.012) and 9-month-old infants (p = 0.006) was associated with a mean difference of ≈0.3 SD in height-for-age *Z*-score at 2 years of age in a study conducted in Pakistan (*45*). Any giardiasis before 2 years of age was a predictor of lower social and intelligence quotients and poorer growth at 3 years of age compared with children without giardiasis in a study from southern India (*42*). Moreover, giardiasis during a child’s first 2 years is associated with cognitive impairment up to 7 years later, independent of physical growth (*43*). Furthermore, a longitudinal study in Peru following ≈140 children from birth to age 9 years describes lower intelligence quotient scores in children with >1 episode of giardiasis annually, compared with children with 1 or no infection (*43*). Of note, most studies examining the association between giardiasis and long-term pediatric implications including stunting, failure to thrive, and cognitive impairment are conducted in low-income or lower middle-income countries, highlighting an important potential health disparity. Higher *G. duodenalis *infection endemicity in lower-resource settings may be contributing to the higher prevalence of stunting and other developmental conditions in these areas.

## Other Complications of Giardiasis

We also surveyed research on additional complications of giardiasis. Those include rarely described complications (e.g., myocarditis and pancreatic neoplasms) and treatment-refractory giardiasis (Appendix).

## Future Directions

The body of literature on giardiasis-associated postinfection sequelae is growing. However, this information largely has been gathered within the context of focal epidemiologic studies or as a follow-up to outbreak investigations, having limited sample sizes, varying clinical criteria, and varying methodologic approaches. Several open areas of research exist to guide giardiasis-associated postinfection sequelae strategies and actions at a national level for the United States.

Of note, a gap exists in research to elucidate the pathophysiology and establish specific diagnostic criteria for IACCIs after giardiasis. Variation in the definitions used to characterize IACCIs complicate the ability to draw definitive conclusions across studies or generate accurate prevalence estimates. Furthermore, given the differences between adult and pediatric clinical manifestations, characterizing the mechanisms of illness at different stages of life may be useful. From the studies we described, we observed a high number of reports of IACCIs after giardiasis based in resource-limited countries. We have an opportunity to understand the biology of how underlying health status, including nutritional, chronic stress, and immunologic profiles, may predispose populations to IACCIs after giardiasis.

In the United States, national-level epidemiologic data on IACCIs after giardiasis are lacking. Routine longitudinal analyses of national hospitalization and insurance claims databases, such as studies presented by Nakao et al. (*22*) and Painter et al. (*33*), may help to identify temporal or regional patterns of IACCIs after giardiasis, including the characterization of high-risk populations by age, sex, race/ethnicity, and insurance type (*22*,*33*). Separately, surveillance activities such as the Foodborne Diseases Active Surveillance Network offer opportunities for state health departments to identify and recruit persons with giardiasis into a prospective cohort study to detect postinfection sequelae and understand risk factors in nonoutbreak settings. As of 2024, however, giardiasis is not among the 8 enteric diseases included in the system. The lack of exposure and symptomatologic data within national surveillance systems contributes to gaps in epidemiologic knowledge for giardiasis. Increasing the robustness of this data collection would enhance our understanding of disease risk factors and clinical manifestations, in turn supporting national prevention and mitigation strategies, identifying populations for targeted outreach, and identifying opportunities for HCP education (*46*).

Finally, research describing HCP awareness and practice regarding IACCIs after giardiasis is lacking, and studies suggest that limited knowledge of giardiasis may be a contributor to illness burden in the United States. Two studies reported gaps in HCP knowledge of giardiasis as a source of childhood diarrhea in the United States (*47*,*48*). Another study found that less than half of surveyed HCPs considered postinfectious IBS as a diagnosis in patients with recent gastrointestinal infections (*49*). With respect to case management, 1 study reported 20% of persons with giardiasis had not received appropriate treatment (*28*), whereas another found that adults often had >3 visits to an HCP before giardiasis diagnosis, were less likely to have a *Giardia*-specific test, and were more likely to receive only antibiotics as treatment (*50*). Those knowledge gaps among HCPs suggest IACCIs after giardiasis probably are underdiagnosed. Resource development to enhance HCP knowledge and aid diagnosis and treatment strategies may help improve case management of affected persons, thereby minimizing the burden of IACCIs and enhancing patient outcomes.

## Conclusions

*G. duodenalis *IACCIs negatively affect the health and quality of life of those in endemic and nonendemic countries (*2*,*6*,*7*). Some priority areas for future progress include creating a consensus around clinical criteria and definitions for IACCIs after giardiasis and increasing data collection through surveillance systems for robust epidemiologic studies. In addition, research is needed to fully understand the underlying mechanisms, national prevalence, populations at increased risk, and specific knowledge gaps for HCPs that may result in delayed diagnosis or misdiagnosis of IACCIs after giardiasis. Ultimately, increased epidemiologic knowledge and educational resources for IACCIs after giardiasis among HCPs may guide public health efforts and contribute to an increased understanding of IACCIs more broadly.

Appendix Additional information about postinfectious syndromes and long-term sequelae after *Giardia* infections.
