# Supplementary material for: Postinfectious Syndromes and Long-Term Sequelae after Giardia Infections
Source: Emerg Infect Dis. 2025 Dec;31(Suppl 2):S45–52. doi: 10.3201/eid3114.241793 (PMC12829484; doi:10.3201/eid3114.241793)
Supplement: Appendix — Additional information about postinfectious syndromes and long-term sequelae after Giardia infections. [file 24-1793-Techapp-s1.pdf]

Article DOI: https://doi.org/10.3201/eid3114213.241793

EID cannot ensure accessibility for supplementary materials supplied by authors. Readers who have difficulty accessing supplementary content should contact the authors for assistance.

Postinfectious Syndromes and Long-Term Sequelae after *Giardia* Infections

Appendix

Other Complications of Giardiasis

Possible Rare Complications of Giardiasis

In addition to the post-infection sequelae described above, there have been case reports describing rare complications involving the heart and pancreas. At least three individuals have been diagnosed with giardiasis-associated myocarditis (*1*–*3*). Two had extensive eosinophilic infiltration in the myocardium (*1*,*2*). As *Giardia* is unable to invade the intestinal mucosa, the authors proposed that these illnesses may have been related to an autoimmune-mediated process in which the immune system attacked the myocardium because of antigenic mimicry (*1*–*3*). Of note, all three cases of myocarditis occurred in the setting of an active *Giardia* intestinal infection. It is unclear whether there may be long-lasting autoimmune implications that could follow treatment and resolution of giardiasis infections.

Other authors have suggested a possible association between giardiasis and gastrointestinal cancers. Although this could be coincidental, several cases describe concurrent giardiasis and new diagnoses of pancreatic neoplasms (*4*–*7*). There is not yet a clear understanding of how the two conditions may be related. It is possible that *Giardia* infections play a role in carcinogenesis, that patients with undiagnosed neoplasms may be at increased risk of giardiasis, or that symptomatic giardiasis infections can lead to incidental detection of existing gastrointestinal cancers.

Treatment-Refractory Giardiasis

When discussing IACCIs following giardiasis, particularly PI-IBS and PI-FD, it is important to consider conditions that can occur even after treatment is administered. While most cases of acute giardiasis resolve spontaneously after two to six weeks of symptoms or sooner

with treatment, infections can persist and are very challenging to treat. Some individuals with suspected PI-IBS may have treatment-refractory giardiasis, as symptoms can mimic one another (*8*). One study followed 124 persons who had one to four courses of conventional treatment with metronidazole yet continued to experience gastrointestinal symptoms. The cohort underwent duodenal biopsies and blood and stool tests. Of those followed, 32% (n = 40) were diagnosed with treatment-refractory giardiasis (*9*). Other studies have identified young age (< 2 years), lower metronidazole doses for initial treatment, comorbidities such as HIV infection, and travel to endemic countries as possible risk factors for treatment-refractory giardiasis (*10*–*12*). Treatment strategies for metronidazole-refractory giardiasis include the use of another single agent (e.g., nitazoxanide or paromomycin) or combination therapy, using two drugs with different mechanisms of action (*13*,*14*). Several studies support quinacrine as an effective single agent, achieving cure in up to 100% of individuals with treatment-refractory giardiasis; however, challenges with quinacrine access and an unfavorable side effect profile often limit its clinical use (*15*–*17*).

References

1. Avşar S, Öz A, Çınar T, Ösken A, Güvenç TS. Acute fulminant eosinophilic myocarditis due to *Giardia lamblia* infection presented with cardiogenic shock in a young patient. Anatol J Cardiol. 2019;21:234–5. PubMed https://doi.org/10.14744%2FAnatolJCardiol.2019.48742

2. Dzierwa K, Rubiś P, Rudnicka-Sosin L, Tekieli L, Pieniążek P. Eosinophilic myocarditis: *Gardia lamblia* infestation and *Garcinia cambogia*. Coincidence or causality? J Rare Cardiovasc Dis. 2016;2:231–5. https://doi.org/10.20418/jrcd.vol2no7.245

3. Robaei D, Vo-Robaei L, Bewes T, Terkasher B, Pitney M. Myocarditis in association with *Giardia intestinalis* infection. Int J Cardiol. 2014;177:e142–4. PubMed https://doi.org/10.1016/j.ijcard.2014.09.050

4. Furukawa M, Lee L, Ikegami T, Maeda T, Nishiyama K, Itaba S, et al. Giardiasis in the pancreas accompanied by pancreatic cancer. Pancreas. 2011;40:168–9. PubMed

5. Hurník P, Žiak D, Dluhošová J, Židlík V, Šustíková J, Uvírová M, et al. Another case of coincidental *Giardia* infection and pancreatic cancer. Parasitol Int. 2019;71:160–2. PubMed https://doi.org/10.1016/j.parint.2019.04.013

6. Kurita A, Maguchi H, Takahashi K, Katanuma A, Osanai M, Kin T, et al. Small pancreatic cancer with giardiasis: a case report. Pancreas. 2010;39:943–5. PubMed https://doi.org/10.1097/mpa.0b013e3181d7883c

7. Mitchell CM, Bradford CM, Kapur U. *Giardia lamblia* trophozoites in an ultrasound-guided fine-needle aspiration of a pancreatic mucinous neoplasm. Diagn Cytopathol. 2011;39:352–3. PubMed https://doi.org/10.1002/dc.21425

8. Berumen A, Edwinson AL, Grover M. Post-infection irritable bowel syndrome. Gastroenterol Clin North Am. 2021;50:445–61. PubMed https://doi.org/10.1016/j.gtc.2021.02.007

9. Hanevik K, Hausken T, Morken MH, Strand EA, Mørch K, Coll P, et al. Persisting symptoms and duodenal inflammation related to *Giardia duodenalis* infection. J Infect. 2007;55:524–30. PubMed https://doi.org/10.1016/j.jinf.2007.09.004

10. Ara-Montojo MF, Bustamante J, Sainz T, Pérez S, Jiménez-Moreno B, Ruiz-Carrascoso G, et al. Intestinal giardiasis in children: five years' experience in a reference unit. Travel Med Infect Dis. 2021;42:102082. PubMed https://doi.org/10.1016/j.tmaid.2021.102082

11. Debnath A, Reed SL, Morris SR. Predictors of failure from primary therapy for giardiasis in San Diego: a single institution retrospective review. Pathogens. 2019;8:165. PubMed https://doi.org/10.3390/pathogens8040165

12. Peters TE, Kreuels B, Addo MM, Tannich E, Rothe C. Risk factors for and management of metronidazole-refractory giardiasis in international travellers: a retrospective analysis. Travel Med Infect Dis. 2021;43:102090. PubMed https://doi.org/10.1016/j.tmaid.2021.102090

13. Carter ER, Nabarro LE, Hedley L, Chiodini PL. Nitroimidazole-refractory giardiasis: a growing problem requiring rational solutions. Clin Microbiol Infect. 2018;24:37–42. PubMed https://doi.org/10.1016/j.cmi.2017.05.028

14. Mørch K, Hanevik K. Giardiasis treatment: an update with a focus on refractory disease. Curr Opin Infect Dis. 2020;33:355–64. PubMed https://doi.org/10.1097/qco.0000000000000668

15. Neumayr A, Schunk M, Theunissen C, Van Esbroeck M, Mechain M, Hatz C, et al. Efficacy and tolerability of quinacrine monotherapy and albendazole plus chloroquine combination therapy in nitroimidazole-refractory giardiasis: a TropNet study. Clin Infect Dis. 2021;73:1517–23. PubMed https://doi.org/10.1093/cid/ciab513

16. Requena-Méndez A, Goñi P, Rubio E, Pou D, Fumadó V, Lóbez S, et al. The use of quinacrine in nitroimidazole-resistant *Giardia duodenalis*: an old drug for an emerging problem. J Infect Dis. 2017;215:946–53. PubMed https://doi.org/10.1093/infdis/jix066

17. Ydsten KA, Hellgren U, Asgeirsson H. Quinacrine treatment of nitroimidazole-refractory giardiasis. J Infect Dis. 2022;225:1773–6. PubMed https://doi.org/10.1093/infdis/jiab287

Supporting References Not Cited in Text

Beatty JK, Akierman SV, Motta JP, Muise S, Workentine ML, Harrison JJ, et al. *Giardia duodenalis* induces pathogenic dysbiosis of human intestinal microbiota biofilms. Int J Parasitol. 2017;47:311–26. PubMed https://doi.org/10.1016/j.ijpara.2016.11.010

Buret AG. Enteropathogen-induced microbiota biofilm disruptions and post-infectious intestinal inflammatory disorders. Curr Trop Med Rep. 2016;3:94–101. https://doi.org/10.1007/s40475-016-0079-x

Buret AG, Amat CB, Manko A, Beatty JK, Halliez MCM, Bhargava A, et al. *Giardia duodenalis*: new research developments in pathophysiology, pathogenesis, and virulence factors. Curr Trop Med Rep. 2015;2:110–8. https://doi.org/10.1007/s40475-015-0049-8

Cantey PT, Roy S, Lee B, Cronquist A, Smith K, Liang J, et al. Study of nonoutbreak giardiasis: novel findings and implications for research. Am J Med. 2011;124:1175.e1–8. PubMed https://doi.org/10.1016/j.amjmed.2011.06.012

Conners EE, Miller AD, Balachandran N, Robinson BM, Benedict KM. Giardiasis outbreaks—United States, 2012–2017. MMWR Morb Mortal Wkly Rep. 2021;70:304–7. PubMed https://doi.org/10.15585/mmwr.mm7009a2

National Academies of Sciences Engineering and Medicine. Toward a common research agenda in infection-associated chronic illnesses: proceedings of a workshop. Snair M, Liao J, Ashby E, Biffl C, editors. Washington: National Academies Press; 2024. https://doi.org/10.17226/27462

Porter CK, Thura N, Riddle MS. Quantifying the incidence and burden of postinfectious enteric sequelae. Mil Med. 2013;178:452–69. PubMed https://doi.org/10.7205/milmed-d-12-00510

Schnell K, Collier S, Derado G, Yoder J, Gargano JW. Giardiasis in the United States—an epidemiologic and geospatial analysis of county-level drinking water and sanitation data, 1993–2010. J Water Health. 2016;14:267–79. PubMed https://doi.org/10.2166/wh.2015.283
